# Relationship between Oversulfation and Conformation of Low and High Molecular Weight Fucoidans and Evaluation of Their *in Vitro* Anticancer Activity

**DOI:** 10.3390/molecules16010291

**Published:** 2010-12-30

**Authors:** Myoung Lae Cho, Boo-Yong Lee, SangGuan You

**Affiliations:** 1 Department of Marine Food Science and Technology, Gangneung-Wonju National University, Gangneung, Gangwon 210702, Korea; E-Mail: meanglae@naver.com (M.L.C.); 2 Department of Food Science and Biotechnology, CHA University, Seongnam, Kyunggi 463836, Korea

**Keywords:** fucoidans, oversulfation, molecular conformation, anticancer activity, *Undaria pinnatifida*

## Abstract

Low and high molecular weight fucoidans (F_5-30K_ and F_>30K_) were chemically modified through the addition of sulfate groups, and the effect of oversulfation on the *in vitro* anticancer activity was investigated. After the addition of sulfate groups, a considerable increase of 35.5 to 56.8% was observed in the sulfate content of the F_5-30K_ fraction, while the sulfate content of the F_>30K_ fraction increased to a lesser extent (from 31.7 to 41.2%). Significant differences in anticancer activity were observed between the oversulfated F_5–30K_ and F_>30K_ fractions, with activities of 37.3–68.0% and 20.6–35.8%, respectively. This variation in the anticancer activity of oversulfated fucoidan derivatives was likely due to differences in their sulfate content. The results suggest that the molecular conformation of these molecules is closely related to the extent of sulfation in the fucan backbones and that the sulfates are preferably substituted when the fucoidan polymers are in a loose molecular conformation.

## 1. Introduction

Fucoidan, a sulfated-fucan, is commonly found in brown seaweed [1] and marine invertebrates, particularly in echinoderms such as sea cucumbers and sea urchins [2]. Fucoidans have been extensively studied due to their diverse anticoagulant, antiviral and anticancer properties. These biological activities have been reported to be closely related to their molecular weight and sulfate content. In a study of fucoidan hydrolysates, partially hydrolyzed fucoidans (M_w_ = 490 kDa) showed significantly improved anticancer activity compared to the native polymers (M_w_ = 5,100 kDa), and the effect was mostly seen when fucoidans were hydrolyzed under mild conditions [3]. The mechanisms whereby lower molecular weight fucoidans have greater anticancer activities appear to be very complex and are not clearly understood. Sulfate content was first identified as a possible factor by Soeda *et al*. [4], who reported that fucoidan derivatives with various sulfate contents stimulate tissue plasminogen activator (t-PA)-induced plasma clot lysis and prevent the formation of fibrin polymers, and that such activities increase proportionally with the degree of sulfation. It was also suggested that oversulfated fucoidans possess higher anti-angiogenic activity than native fucoidans, and thus more effectively inhibit the growth of tumor cells by suppressing angiogenesis [5]. Partially desulfated fucoidans with sulfate contents of less than 20%, on the other hand, show drastic decreases in both anticoagulant and anticancer activities [6]. These studies suggest that the biological activities of fucoidans are significantly influenced by sulfate content and could be improved by the modification of this feature. Numerous studies have investigated the biological activities of oversulfated fucoidans. However, the extent of sulfation following the oversulfation reaction has been variable, ranging from 48 to 56% [5,7,8]. Despite these differences, there have been no reports of the factors affecting the extent of sulfation of fucoidans.

In a preliminary study, we extracted a fucoidan from the sporophyll of *Undaria pinnatifida*, hydrolyzed it under a mild acidic conditions and subsequently fractionated it using an ultrafiltration system equipped with 30 and 5 kDa membranes, which resulted in the production of three fucoidan fractions: F_>30K_, F_5-30K_ and F_<5K_. The major constituents of the F_>30K_ and F_5–30K_ fractions were carbohydrate (58.2–61.3%) and sulfate (31.7–35.5%) with a small amount of protein, while that of the F_<5K_ fraction was mostly sulfate and ash with little carbohydrate (15.5%). When the *in vitro* anticancer activities of these fractions were studied, the F_5–30K_ fraction exhibited the most potent anticancer activity. In the current study, the F_>30K_ and F_5–30K_ fractions (having molecular weights of 262 and 5.6 kDa, respectively), were chemically modified by the addition of sulfate groups, and the effect of the molecular conformation on the extent of sulfation and the *in vitro* anticancer activity of oversulfated fucoidan derivatives was investigated.

## 2. Results and Discussion

The sulfate contents of two native fucoidan fractions and their oversulfated derivatives are shown in Table 1. After the addition of sulfate groups, there was a considerable increase in the sulfate content of the F_5-30K_ fraction from 35.5% to 56.8%. In contrast, the sulfate content of the F_>30K_ fraction only increased from 31.7% to 41.2%.

**Figure 1:**
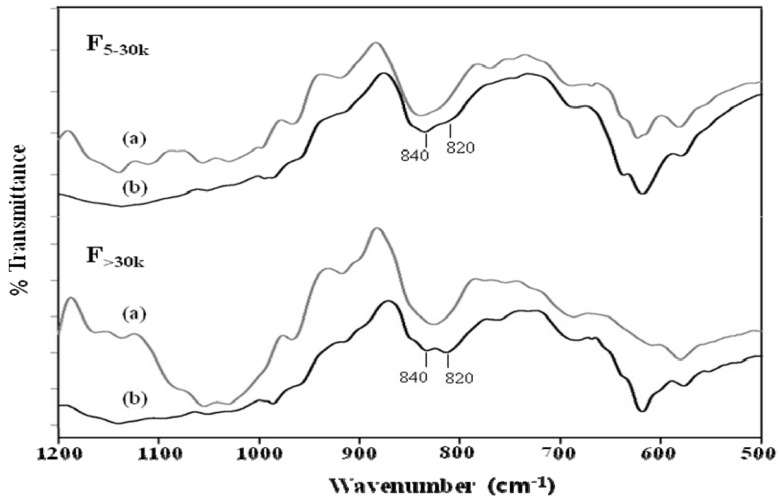
FT-IR spectrums of (a) native and (b) oversulfated fucoidans. The fucoidans were scanned between 500 and 1,200 cm^−1^.

These changes in sulfate content were also supported by evidence from the IR spectra (Figure 1). The sulfate peaks around 820 and 840 cm^−1^ are related to the equatorial C-2 and axial C-4 positions, respectively [9]. As shown in Figure 1, the native fucoidans of the F_5-30K_ and F_>30K_ fractions exhibited a strong peak around 840 cm^−1^, indicating that the sulfates were largely substituted at the C-4 position. In contrast, the oversulfated derivatives of F_5–30K_ and F_>30K_ fractions displayed a major peak at 840 cm^−1^ with a shoulder at 820 cm^−1^, indicating the 2,4 disubstitution of sulfate groups. These results are consistent with those of Qiu *et al*. [8], who also reported that sulfation led to the appearance of a shoulder at 820 cm^−1^ accompanied by a main peak at 840 cm^−1^ in the IR spectra, suggesting the occurrence of the 2,4 disulfation. Sulfation at the C-2 and/or C-4 positions is most likely to occur if the backbones of the fucoidans extracted in this study were connected by 1,3-glycosidic bonds. 

**Figure 2:**
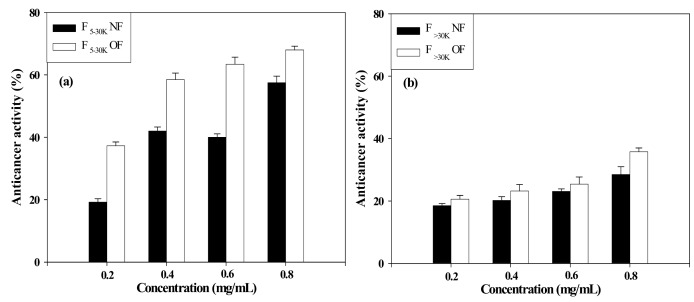
Anticancer activity of native and oversulfated fucoidans against the human stomach cancer cell line AGS. (a) F_5-30K_ fraction of native and oversulfated fucoidans and (b) F_>30K_ fraction of native and oversulfated fucoidans.

The anticancer activity of native and oversulfated fucoidans, expressed as a percentage of the growth inhibition of the cancer cell line AGS, is shown in Figure 2. At concentrations from 0.2 to 0.8 mg/mL, the native F_5-30K_ fraction inhibited the growth of cancer cells in a strong dose-dependent manner and had anticancer activities in the range of 19.2% to 57.5%. In contrast, the native F_>30K_ fractions displayed a relatively weak dose-dependent inhibition and had anticancer activities in the range of 18.0% to 28.5%. When oversulfated, the fucoidan derivatives significantly increased the inhibition of cell growth. Significant differences were observed in the anticancer activities of the oversulfated F_5–30K_ and F_>30K_ fractions, with the F_5–30K_ fraction inhibiting growth by 37.3% to 68.0% and the F_>30K_ fraction by 20.6% to 35.8%, depending on the fucoidan concentration. This considerable variation in the anticancer activity of the oversulfated fucoidan derivatives is likely due to differences in their sulfate content. This finding is supported by the results of Haroun-Bouhedja *et al*. [6], who showed that the antiproliferative activity of CCL39 cells proportionally increases with sulfate content and that fucoidans containing more than 40% sulfates inhibit the growth of CCL 39 cells by over 90%. Although the underlying mechanism was not completely understood, the authors suggested that increased negative charges caused by oversulfation facilitate the formation of fucoidan-protein complexes involved in the cell proliferation, resulting in a greater suppression of cell growth. 

Our results suggest that the extent of sulfation can vary between fucoidan samples during the sulfation reaction, probably because of differences in their molecular structures such as molecular weight and conformation. In our preliminary study, when molecular weight (M_w_) was plotted against the radius of gyration (R_g_), based on the following relationship: R_g_ = KM_w_^α^ (sphere if α < 0.3; random coil if 0.3 ≤ α < 0.5; rod if α ≥ 0.5) [10], the native F_>30K_ fraction appeared to exist in a very compact conformation (α = 0.15), possibly due to intra-molecular interactions, while the native F_5-30K_ fraction appeared in a random coil conformation (α = 0.42). As shown in Table 1, after the sulfation reaction, the sulfate content of the F_5-30K_ fraction increased to a greater extent than that of the F_>30K_ fraction (21.3% *vs.* 9.5%, respectively), suggesting that molecular conformation might affect the extent of sulfation in the fucoidan backbones during the sulfation reaction. The compact spherical conformation of the F_>30K_ fraction likely indicates that the hydroxyl groups available for the substitution of sulfates were located inside the chains and might be involved in intramolecular interactions. Consequently, the compact conformation of the F_>30K_ fraction would limit the degree of sulfation. However, for the F_5–30K_ fraction, the fucoidan polymers were in a loose conformation, with more hydroxyl groups likely exposed and available for sulfate substitution. Therefore, the somewhat less compact conformation of the F_5–30K_ fraction might result in the extensive sulfation of the fucan backbones and produce more highly sulfated fucoidan derivatives with improved anticancer activity. Despite the numerous studies on oversulfated fucoidans, the correlation between the extent of sulfation and the molecular conformation of fucoidans has not been reported. However, in the sulfation study of heparan polymers, it was suggested that a conformational arrangement of the polymeric chain would play an important role in the extent of sulfation because the exposure of the available hydroxyl groups would influence the degree of sulfate substitution [11]. 

## 3. Experimental

### 3.1. Extraction, hydrolysis and fractionation of fucoidan

The dried sporophyll of brown seaweed (*Undaria pinnatifida*), originating from the coast of Wando, Chunnam province, Korea, was purchased, milled using a blender, sieved (<0.5 mm) and then stored at −20 °C until analysis. Native fucoidan was extracted from the dried biomass as previously reported [3]. Briefly, the dried biomass (5 g) was rehydrated in distilled water (200 mL) at 65 °C for 1 h. The soluble solid extract was mixed with 1% CaCl_2_ and precipitated by centrifugation to remove alginic acid. Ethanol (99%, 500 mL) was added to the supernatant to obtain a final ethanol concentration of 70% (v/v), and then the native fucoidan was filtered from the ethanol solution. 

The native fucoidan was hydrolyzed according to the method of Nardella *et al*. [12] to produce low molecular weight fucoidans. The hydrolysis of fucoidan was carried out by dissolving the native fucoidan (1.0 g) and copper acetate monohydrate mixture (0.08 g, 0.04 mM) in water (10 mL) at 60 °C while maintaining the pH at 7.5 by the addition of 2M NaOH (0.2 mL). Hydrogen peroxide solution (90%, v/v, 0.5 mL) was added to the fucoidan mixture at a flow rate of 12 mL/h, and hydrolysis by copper acetate monohydrate was performed for 5 hr with constant mechanical stirring. The hydrolyzed fucoidan was fractionated using a Millipore Ultrafiltration System with 30 and 5 kDa molecular weight cut-off membranes (0.1 m^2^, Millipore, USA). This produced three fucoidan fractions, F_>30K_ (M_w_ > 30kDa), F_5–30K_ (30kDa<M_w_ <5kDa) and F_<5K_ (M_w_ <5kDa). Oversulfated fucoidan was obtained using the dimethylformamide (solvent) and sulfur trioxide–trimethylamine complex (sulfating agents) according to the method of Soeda *et al*. [4].

### 3.2. Proximate composition and in vitro anticancer activity

The sulfate content of fucoidan was determined by the BaCl_2_ gelatin method using K_2_SO_4_ as a standard after hydrolyzing the polysaccharide in 0.5 M HCl at 105 °C for 5 hr [13]. Total carbohydrate and total protein were determined by the phenol-sulfuric acid method using fucose as a standard [14] and by the Lowry method [15] using a DC Protein assay kit (Bio-Rad, Hercules, CA, USA), respectively. The FT-IR spectra (KBr disc) were recorded using a Tensor 27 spectrophotometer (Bruker, Germany). The anticancer activity of fucoidans was determined using the sulforhodamine B (SRB) assay, as previously reported by Yang *et al*. [3]. 

## 4. Conclusions

This study describes the importance of molecular conformation on the sulfation of fucoidans, suggesting that the molecular conformation has a close relationship with the extent of sulfation in the fucan backbones during the sulfation reaction, with sulfate substitution occurring preferentially on fucoidans with a loose molecular conformation. In addition, the degree of sulfation appeared to influence the anticancer activity of fucoidans. It would therefore be beneficial to derive fucoidans with a less compact conformation as substrates for the extensive sulfate substitution and for the production of oversulfated fucoidans with greater anticancer activities.

## Figures and Tables

**Table 1:** The sulfate contents of native and oversulfated fucoidans.

Sample	F_5-30k_ (%)	F_>30k_ (%)
Native fucoidan	35.5 ± 1.9	31.7 ± 2.2
Oversulfated fucoidan	56.8 ± 2.3	41.2 ± 0.9
